# Supplementary material for: A low-cost FPGA-based approach for pile-up corrected high-speed in vivo FLIM imaging
Source: Neurophotonics. 2025 May 5;12(2):025009. doi: 10.1117/1.NPh.12.2.025009 (PMC12052397; doi:10.1117/1.NPh.12.2.025009)
Supplement: Supplementary file 1 [file NPh_012_025009_SD001.pdf]

A low-cost FPGA-based approach for pile-up corrected high-speed in vivo FLIM imaging – Supplementary Information

Felipe Velasquez Moros[1,#], Dorian Amiet[2,#,*], Rachel M. Meister[1], Alexandra von Faber-Castell[1], Matthias Wyss[1], Aiman S. Saab[1,3], Paul Zbinden[2], Bruno Weber[1,3], Luca Ravotto[1,*]

[#]These authors contributed equally
[1] Institute of Pharmacology and Toxicology, University of Zurich, Zurich, Switzerland;
[2] IMES Institute for Microelectronics, Embedded Systems and Sensorics, OST - Eastern Switzerland University of Applied Sciences, Rapperswil, Switzerland;
[3] Neuroscience Center Zurich, University and ETH Zurich, Zurich, Switzerland;

[*] Correspondence to: luca.ravotto@pharma.uzh.ch, dorian.amiet@ost.ch

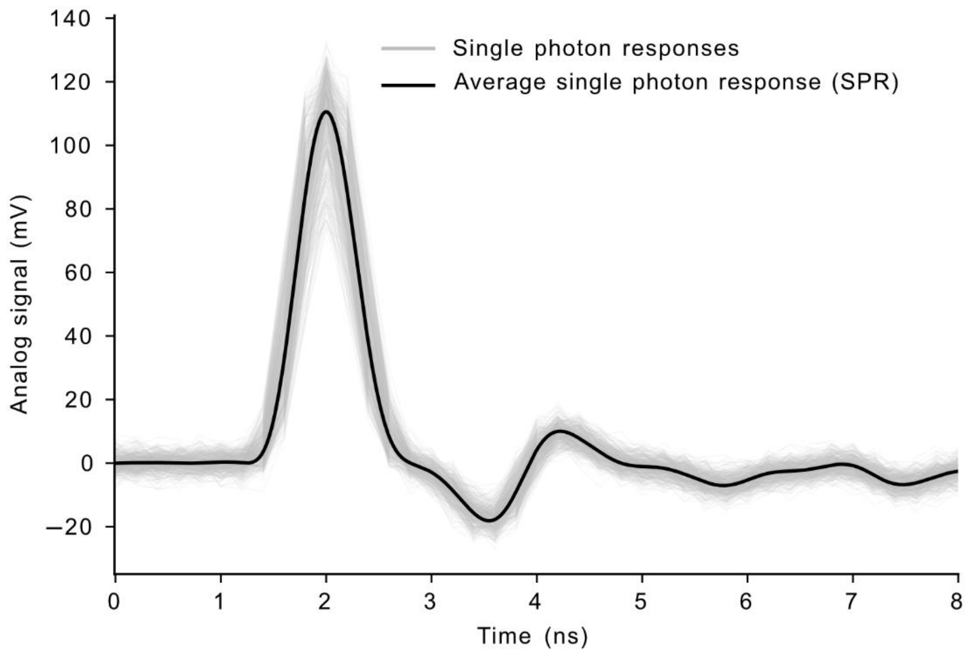

Figure S1: Single-photon response of the PMA-40mod Hybrid Photodetector (PicoQuant, Germany) used in this study.

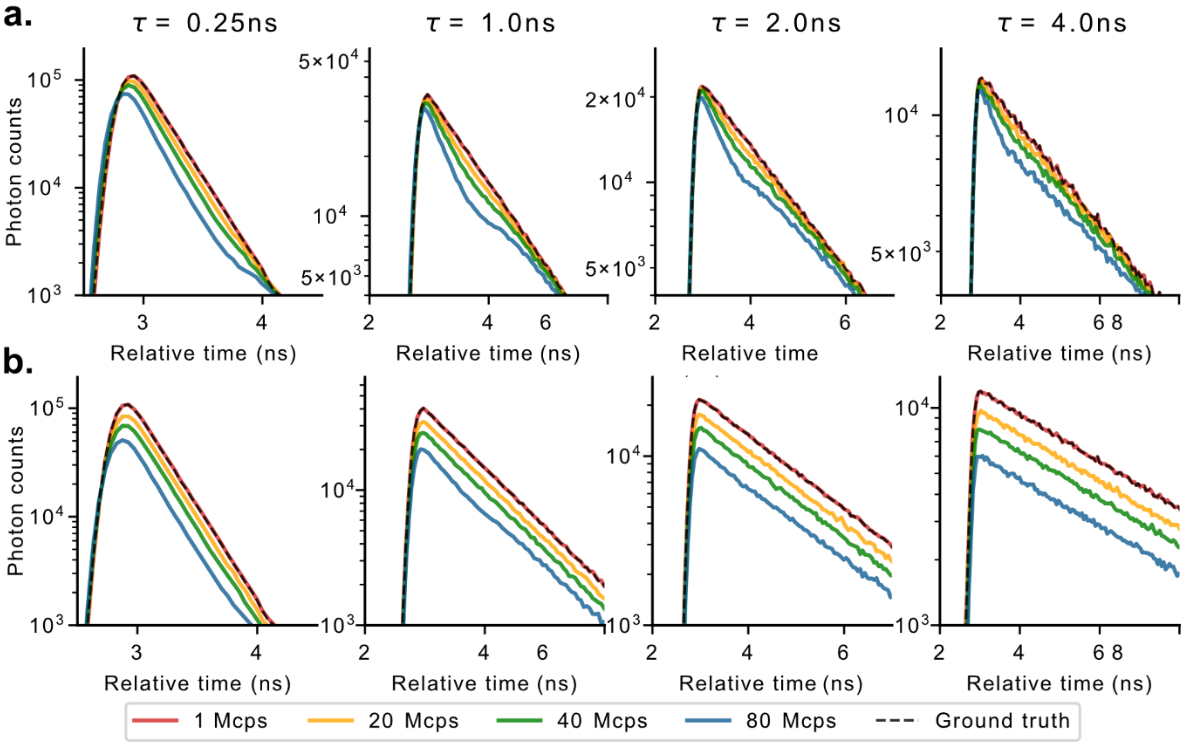

Figure S2: Simulated decays for different monoexponential lifetimes and count rates with the before (a) and after (b) application of the LPBT correction. The SPR shown in Figure S1 was used to simulate the detector response.

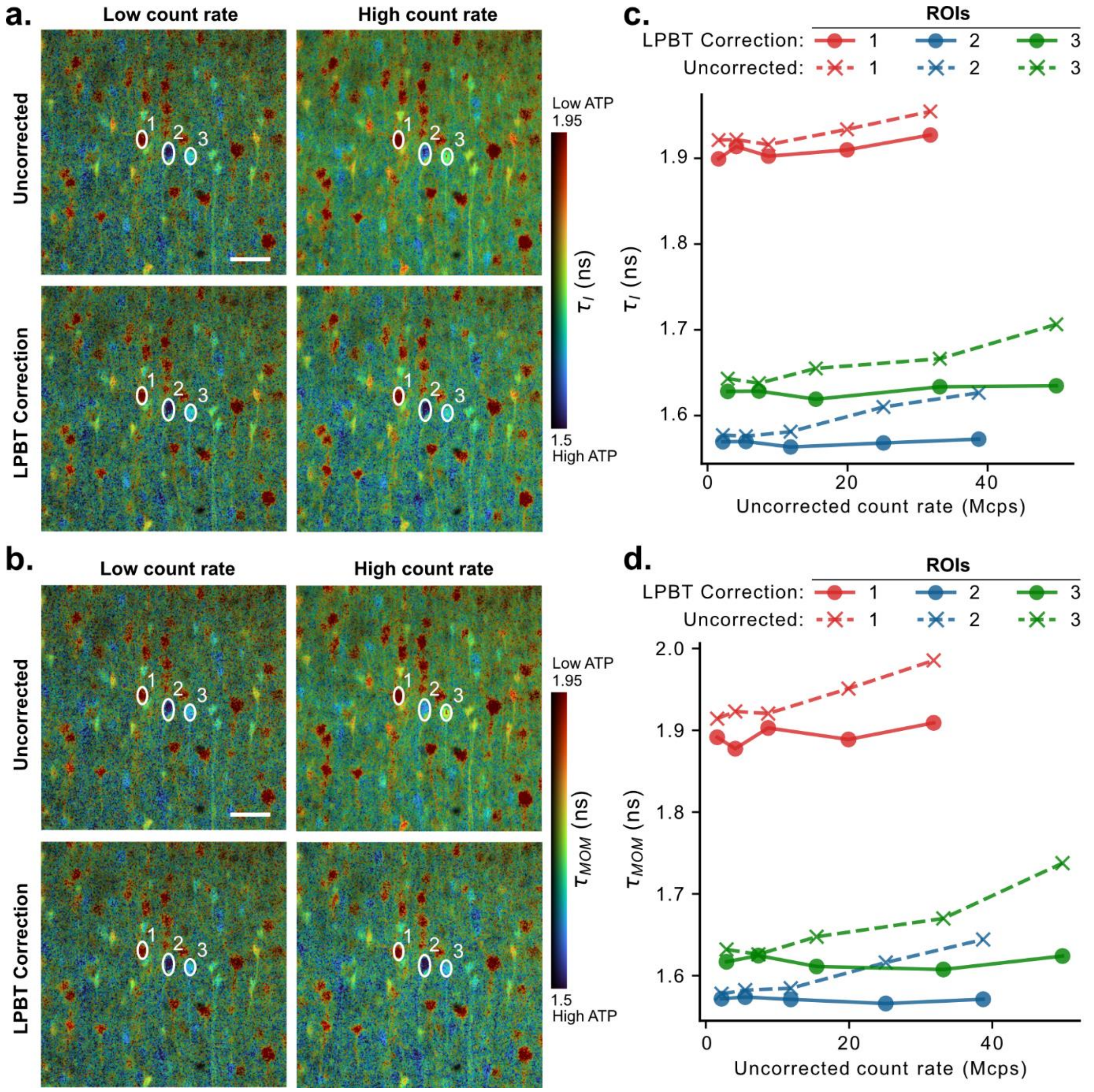

Figure S3: (a,b) Comparison of LPBT corrected and uncorrected (detected) ATP lifetime images at low (<6 Mcps) and high (up to 85 Mcps) count rates and using different lifetime estimators: (a) τ_I and (b) τ_{MOM}. (c, d) The change in τ_I (c) and τ_{MOM} (d) for each selected ROI as the detected count rate increases. Scale bar = $40\ \mu m$.

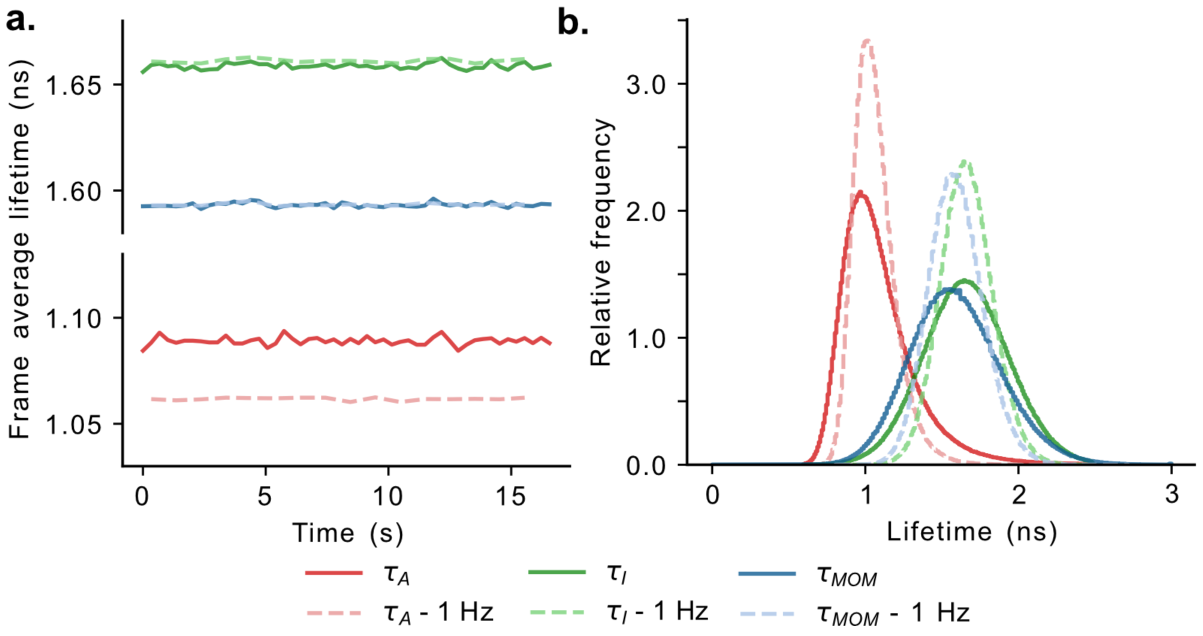

Figure S4: (a) Frame average lifetime at baseline for ATP images using different estimators with and without time binning. (b) Distribution of pixel lifetime values at baseline using different estimators with and without time binning.

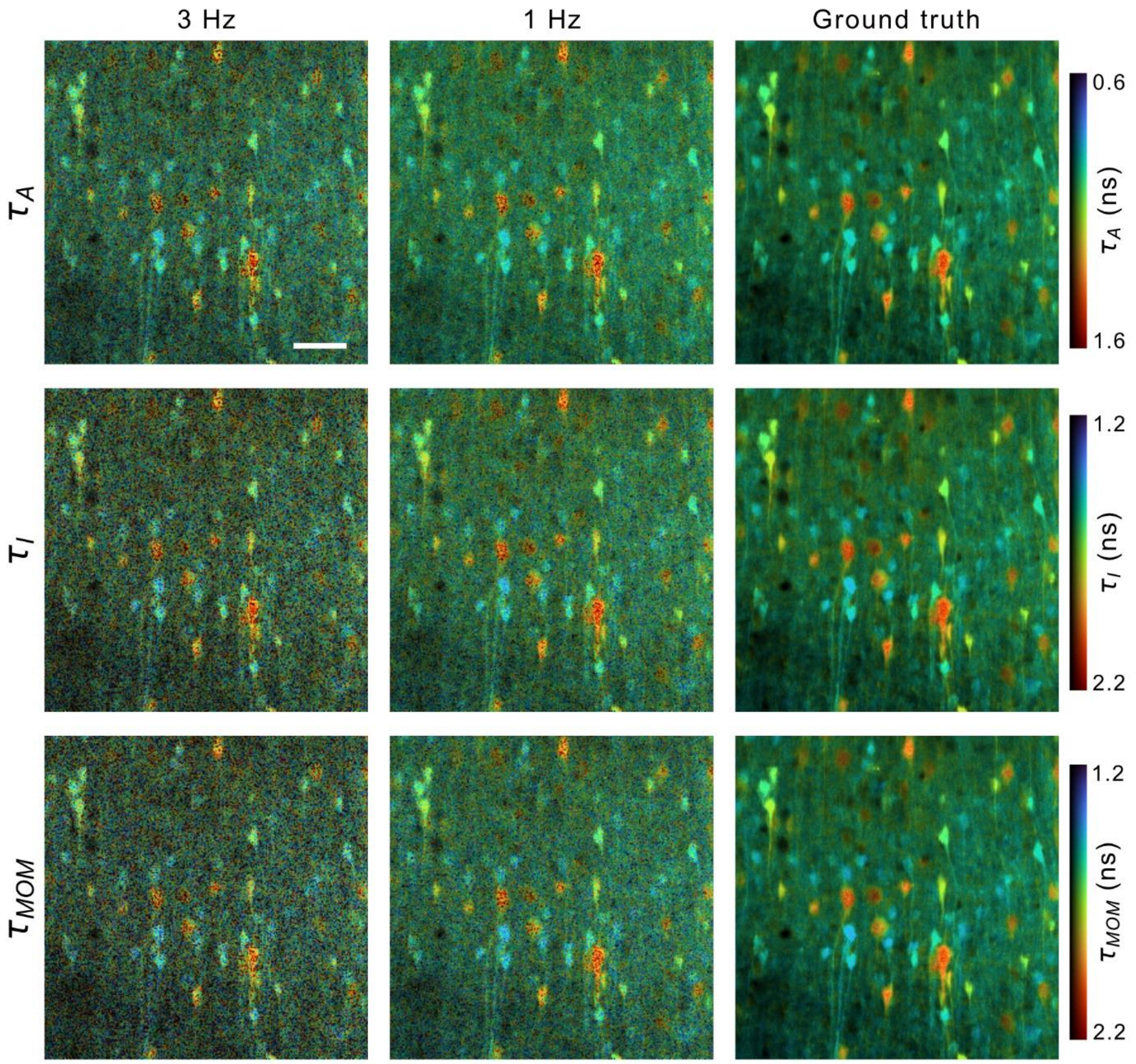

Figure S5: Lifetime images of ATP at baseline for 3 Hz (single frame), 1 Hz (average of three frames), and ground truth (average of 150 frames) for all lifetime estimators at 256x256 pixels resolution. Scale bar = 40 μm.

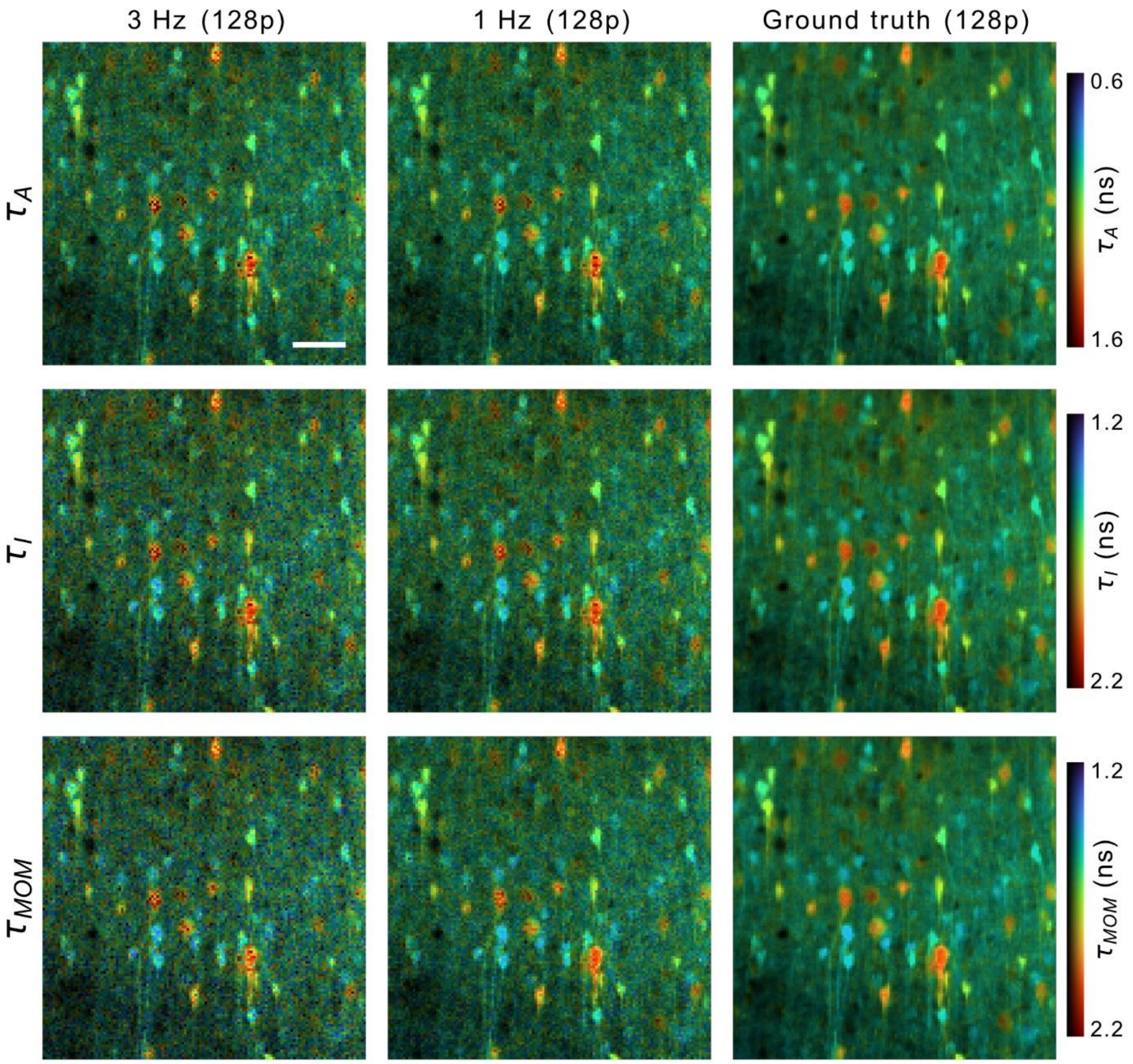

Figure S6: Lifetime images of ATP at baseline for 3 Hz (single frame), 1 Hz (average of three frames), and ground truth (average of 150 frames) for all lifetime estimators at 128x128 pixels resolution. Scale bar = 40 μm.

Supplementary Video 1: Video recording showing the response of the ATP sensor Ateam1.03YEMK to NaN_3 application, at 256x256 resolution. The panel on the left shows the acquired FLIM data at the 3 Hz imaging speed while the panel on the right shows the result of applying temporal binning for every three frames, effectively reducing the imaging speed to 1 Hz. The plot on the bottom shows the method-of-moments lifetime (τ_{MOM}) calculated as average across a frame at 3 Hz. To reduce the video length, the reproduction speed was increased 10 times with respect to the actual imaging speed. Scale bar = 40 μm.

Supplementary Video 2: Video recording showing the response of the ATP sensor Ateam1.03YEMK to NaN_3 application, at 128x128 resolution. The panel on the left shows the acquired FLIM data at the 3 Hz imaging speed while the panel on the right shows the result of applying temporal binning for every three frames, effectively reducing the imaging speed to 1 Hz. The plot on the bottom shows the method-of-moments lifetime (τ_{MOM}) calculated as average across a frame at 3 Hz. To reduce the video length, the reproduction speed was increased 10 times with respect to the actual imaging speed. Scale bar = 40 μm.

Supplementary Note 1: Analysis of TCSPC counting efficiency with pulse pile-up correction methods

Inherent photon loss due to pile-up

When two photons arrive at the detector with a small enough time difference Δt the electrical pulses generated by their detection will overlap. Below a certain Δt, these two piled up photons will be registered as a single event as the electrical signal will cross the detection threshold only once. Therefore, when the probability of pile-up is significant, an increase in the photon rate arriving at the detector does not lead to an equivalent increase in the number of detected TCSPC events. The following derivation shows how the impact of pile-up on TCSPC collection efficiency can be assessed.

The counting efficiency of the system, η, can be defined as:

$$\eta = \frac{CR_{detected}}{CR_{incident}}$$

where $CR_{incident}$ is the average photon rate impinging on the detector, and $CR_{detected}$ is the detected one (in presence of pile-up $\eta < 1$). To estimate η at a given incident photon count rate $CR_{incident}$, it is necessary to take into account the distribution of photon arrival times within an excitation cycle $P_{incident}(t)$ and the average number of photons expected per excitation cycle $\lambda_{in} = CR_{incident}/f_{las}$, where f_{las} is the laser repetition rate.

For simplicity, we model the distribution of arrival times as a monoexponential decay with lifetime τ (the conclusions can be generalized to any decay model), excited by a train of N pulses with excitation period $T_{las} = 1/f_{las}$. The probability distribution for the decay is given by:

$$P_{decay}(t) = \frac{1}{\tau}\sum_{i=0}^{N} e^{-\frac{t+i\cdot T_{las}}{\tau}} \quad \forall\, t \in [0, T_{las})$$

It can be shown that the $\lim_{N\to\infty} \int_0^{T_{las}} P_{decay}(t)dt = 1$, thus, to ensure that $P_{decay}(t)$ is a probability density function, the following analysis is only valid when $N * T_{las} \gg \tau$. This condition is almost always fulfilled in reality as collecting enough photons requires many excitation cycles and usually $\tau < T_{las}$. Furthermore, when this condition is fulfilled $P_{decay}(t)$ is periodic with respect to the excitation cycle: $P_{decay}(t \pm T_{las}) = P_{decay}(t)$.

Then, for a given time period $[t - \Delta t, t]$ within an excitation cycle, the expected number of events is given by:

$$\lambda(t, \Delta t) = \lambda_{in} \int_{t-\Delta t}^{t} P_{decay}(t')dt'.$$

It is important to note that $P_{decay}(t)$ is only defined for $t \in [0, T_{las})$, thus to evaluate this integral when $t - \Delta t < 0$ one must make use of its periodicity and split the integral into two parts:

$$\lambda(t, \Delta t) = \lambda_{in} \left(\int_0^t P_{decay}(t')dt' + \int_{t-\Delta t+T_{las}}^{T_{las}} P_{decay}(t')dt' \right).$$

The minimum Δt between two photons that does not result in pile-up, which we designate here as the blind time of the system (t_{blind}), depends on the temporal response of the detector and the chosen threshold. In order to detect a photon at any given time t there cannot be any incident

photons in the time period $[t - t_{blind}, t]$. Since photon arrivals are independent from each other, the probability of getting a specific number of photons k during a given time period is modeled by the Poissson distribution:

$$Poisson(k) = \frac{\lambda^k e^{-\lambda}}{k!},$$

Where λ is the expectation of the number of events during the given time period. Setting $k = 0$ and $\lambda = \lambda(t, t_{blind})$ yields the probability of having no incident photons during the blind time of the system preceding t: $P_{zero} = e^{-\lambda(t,t_{blind})}$.

In absence of pile-up, the probability to detect the photon $P_{detection}(t)$ is equal to $P_{decay}(t)$, while in presence of pile-up, we must multiply $P_{decay}(t)$ by the probability of the detection system being active $P_{active}(t, \lambda_{in})$:

$$P_{detected}(t, \lambda_{in}) = P_{decay}(t) \cdot P_{active}(t, \lambda_{in})$$

And since $P_{active}(t, \lambda_{in})$ is equal to the probability of not having detected a photon in the time period $[t - t_{blind}, t]$, we can write:

$$P_{detected}\left(t, \lambda_{\,t,t_{blind}}\right) = P_{decay}(t) \cdot e^{-\lambda(t,t_{blind})}$$

The detection efficiency can be calculated as the ratio between the integrals of the detected and incident probabilities over the entire period:

$$\eta = \frac{\int_0^{T_{las}} P_{detected}(t, \lambda(t, t_{blind}))dt}{\int_0^{T_{las}} P_{decay}(t)dt} = \int_0^{T_{las}} P_{detected}(t, \lambda(t, t_{blind}))dt$$

which can be evaluated numerically to obtain the theorethical efficiency curves for diferent values of τ shown in Figure S7.

Photon loss with the LPBT correction

The effect of the LPBT correction on the counting efficiency can be calculated analitically for an ideal case in which the electrical pulse width are infinitely sharp. In this case, the derivation shown above must be modified to account for the fact that any photons that arrive during an imposed blind time will not have any impact on the duration of the blind period. This means $P_{active}(t, \lambda_{in})$ can no longer be defined using the Poisson statistics of incident photons, instead it must be defined with respect to the probability of having detected a photon in the period $[t - t_{blind}, t]$:

$$P_{active}(t, \lambda_{in}) = 1 - \lambda_{in} \int_{t-t_{blind}}^{t} P_{detected}(t')dt'.$$

Imposing the condition of the LPBT correction ($t_{blind} = T_{las}$) and the periodicity of the photon distribution, the integral term in $P_{active}(t, \lambda_{incident})$ can be simplified:

$$\int_{t-T_{las}}^{t} P_{detected}(t')dt' = \int_0^{T_{las}} P_{detected}(t)dt = \eta_{LPBT} \int_0^{T_{las}} P_{decay}(t)dt = \eta_{LPBT}$$

Notably, setting the dead time to match the excitation period removes the dependance of P_{active} on t (and therefore τ), demonstrating the unbiased nature of the correction.

Introducing this back into the $P_{detected}(t, \lambda_{in})$ equation we get:

$$P_{detected}(t, \lambda_{in}) = P_{decay}(t) \cdot (1 - \lambda_{in} \cdot \eta_{LPBT})$$

To get an explicit equation for the collection efficiency of the LPBT correction (η_{LPBT}) we integrate the $P_{detected}(t, \lambda_{in})$ over the excitation period and simplify:

$$\eta_{LPBT} = 1 - \lambda_{in} \cdot \eta_{LPBT}$$

leading to:

$$\eta_{LPBT} = \frac{1}{1 + \lambda_{in}}$$

In our implementation of the LPBT correction, the finite electrical pulse width introduces a slight non-ideality and a slight dependence of the collection efficiency on the lifetime. To estimate the impact of the non-ideality, we extracted the counting efficiency for simulated photon streams using the average pulse shape from a Picoquant PMA-40mod HPD detector. As shown in Figure S7a, the impact is negligible.

High speed FLIM filter (HSFF) correction

To provide a point of comparison for the collection efficiency of our method, we can derive the same parameter for the commercially available method implemented by Leica. The HSSF correction methods discards the data from all excitation periods with more than one photon. Therefore, the average number of detected photons per excitation cycle $\lambda_{detected}$ is given by the probability of having excactly one photon during an excitation cycle. Using the Poisson distribution we obtain:

$$\lambda_{detected} = P(1) = \lambda_{in} \cdot e^{-\lambda_{in}}$$

The collection efficiency is thus:

$$\eta_{HSFF} = \frac{CR_{detected}}{CR_{incident}} = \frac{\lambda_{detected}}{\lambda_{in}} = e^{-\lambda_{in}}$$

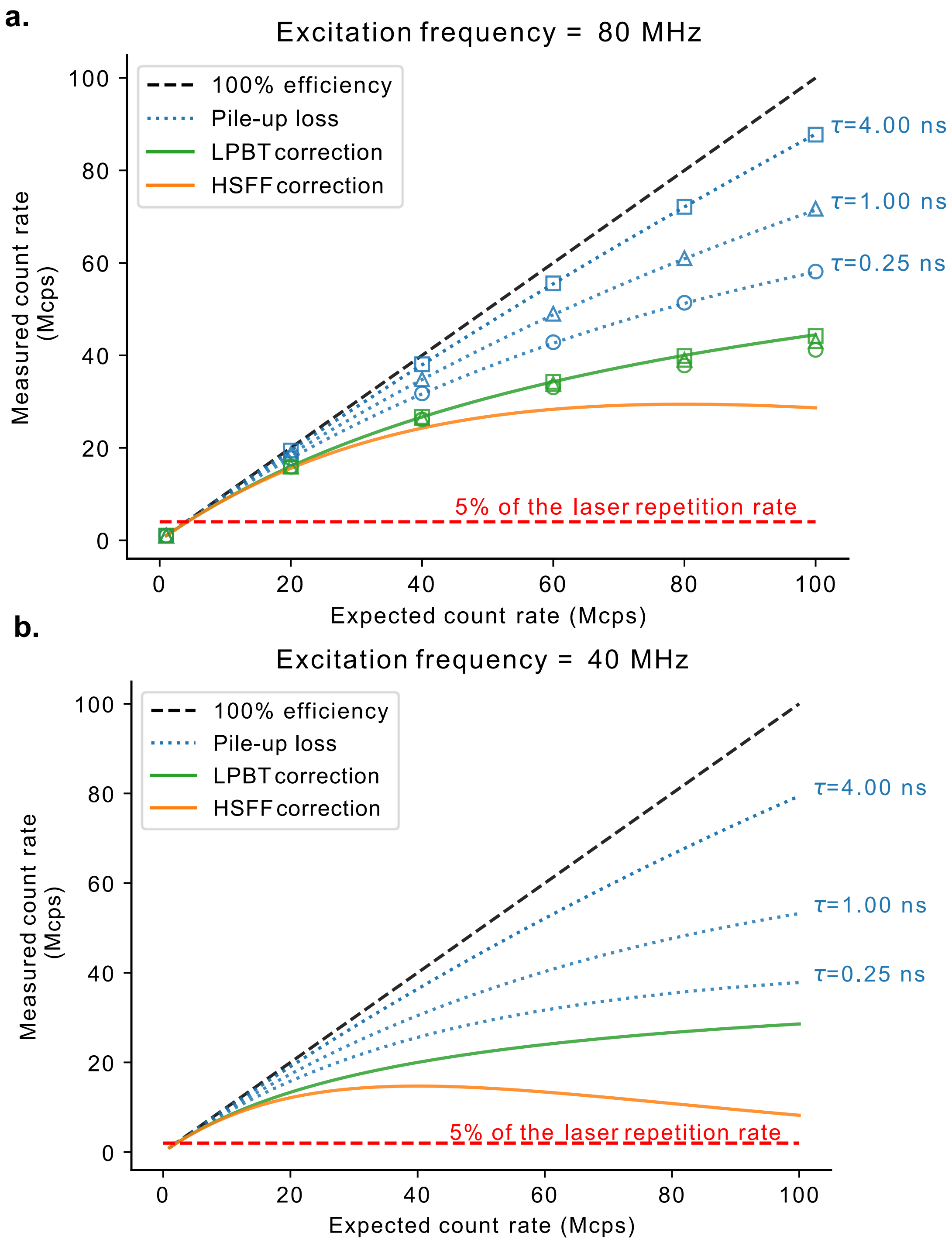

Figure S7: Photon collection efficiency of various TCSPC approaches with 80 MHz (a) or 40 MHZ (b) repetition rate laser. The lines show theoretical efficiencies and the data points are extracted from simulations with different monoexponential lifetimes assuming the average pulse shape from a Picoquant PMA-40mod HPD detector (squares: 4 ns, triangles: 1 ns, and circles: 0.25 ns). The black (dashed) line defines the 100% collection efficiency limit, the blue (dotted) lines show the direct impact of pile-up before any corrections ($t_{blind} = 0.85$ ns), and the green and orange lines show the collection efficieies of the LPBT (ours) and HSFF (commercially available) correction methods. Even though the collection efficiency of the LPBT method is theoretically independent of the lifetime, our non-ideality introduces a small distortion that does depend on the lifetime. The green data points show its impact based on the previously mentioned simulations. Finally, the red dashed line shows the traditional 5% of the laser repetition rate limit.
